# Malaria, anemia, and invasive bacterial disease: A neutrophil problem?

**DOI:** 10.1002/JLB.3RI1018-400R

**Published:** 2018-12-20

**Authors:** Jason P. Mooney, Lauren J. Galloway, Eleanor M. Riley

**Affiliations:** ^1^ The Roslin Institute and Royal (Dick) School of Veterinary Studies University of Edinburgh Midlothian United Kingdom

**Keywords:** malaria, salmonella, anemia, sepsis, neutrophil, heme oxygenase‐1, IL‐10

## Abstract

Invasive bacterial disease is well described in immunocompromised hosts, including those with malaria infection. One bacterial infection frequently observed in children with *Plasmodium falciparum* infection is nontyphoidal *salmonella* (NTS) infection, in which a typically intestinal infection becomes systemic with serious, often fatal, consequences. In this review, we consider the role of malaria‐induced immunoregulatory responses in tipping the balance from tissue homeostasis during malaria infection to risk of invasive NTS. Also, neutrophils are crucial in the clearance of NTS but their ability to mount an oxidative burst and kill intracellular *Salmonella* is severely compromised during, and for some time after, an acute malaria infection. Here, we summarize the evidence linking malaria and invasive NTS infections; describe the role of neutrophils in clearing NTS infections; review evidence for neutrophil dysfunction in malaria infections; and explore roles of heme oxygenase‐1, IL‐10, and complement in mediating this dysfunction. Finally, given the epidemiological evidence that low density, subclinical malaria infections pose a risk for invasive NTS infections, we consider whether the high prevalence of such infections might underlie the very high incidence of invasive bacterial disease across much of sub‐Saharan Africa.

**Abbreviations**
*Hmox1*: heme oxygenase‐1 gene
HO‐1: heme oxygenase‐1
NETs: neutrophil extracellular traps
NTS: nontyphoidal *Salmonella*
PMN: polymorphonuclear cells
ROS: reactive oxygen species
SCV: *Salmonella* containing vacuoles

## 1 BACTEREMIA AND MALARIA

Bloodstream bacterial infections remain a global health concern, with high case fatality rates and the potential for long‐term, life‐changing sequelae. Life‐threatening organ dysfunction resulting from systemic bacterial infection, or more commonly sepsis,^1^ is mediated by a systemic inflammatory response^2^, ^3^ wherein septic shock leads to severe tissue damage and death.^4^, ^5^ Sepsis is one of the most challenging and most costly conditions to treat in hospital—amassing a bill of $24 billion in the United States for 2013 alone.^6^

In developed economies, the organisms most frequently isolated from blood include *Staphylococcus aureus* and *Escherichia coli* 7 (each accounting for ∼20% of cases). Methicillin‐resistant *S. aureus* 8 and highly pathogenic *E. coli* are emerging as major causes of nosocomial infections.^9^ In contrast, developing nations in Africa see a much greater incidence of community‐acquired bacteremia with *Salmonella enterica* (often nontyphoidal *Salmonella* [NTS]) and *Streptococcus pneumoniae* as the most commonly isolated organisms.^10^ Laboratory diagnosis for microbiological pathogens in Africa remains poor, with insufficient infrastructure and related funding. Despite challenges in detection, Ao et al. have estimated that NTS causes 3.4 million cases of bacteremia globally each year, of which the majority (1.9 million cases and 380,000 deaths) are in children and young adults in sub‐Saharan Africa.^11^ In Kenya, 70% of these deaths occur within 2 days of admission to hospital,^12^ providing a very narrow window for effective intervention. Further, multiple drug‐resistant NTS serotypes have been reported in East and Southern Africa, with sequence type 313 (ST313) seen as a distinct lineage associated with septicemia.^13^, ^14^ Increasingly, lack of access to effective and affordable antibiotics may lead to even higher morbidity and mortality in low‐income settings.

NTS thrives in the intestinal environment where, in otherwise healthy hosts, localized gastroenteritis allows NTS to outcompete the microbiota, causing diarrhea and promoting transmission.^15^ However, the infection can “escape” the gut and invade other tissues, eventually becoming systemic, particularly when the host is immunocompromised. One well‐documented risk factor for invasive NTS is *Plasmodium falciparum* malaria.^16^, ^17^ *Plasmodium*, the causative agent of malaria, is transmitted to humans through the bite of the female *Anopheles* mosquito causing a range of clinical manifestations including anemia, metabolic acidosis, and end‐organ failure.^18^ In The Gambia, the incidence of invasive NTS infection mirrors that of malaria, peaking during the annual rainy season, and in one study, 43% of children with *Salmonella* bacteremia had concurrent *P. falciparum* infections.^19^ In Tanzania, invasive NTS in young children is highly associated with recent malaria infection, with 78% of NTS cases having recently received antimalarial medication and 82% of cases being anemic.^20^ Intriguingly, recent (past) malaria infection is a higher risk factor for NTS bacteremia than is acute (current) infection.^21^ Therefore, although children with severe acute malaria have been noted to be at high risk of developing invasive NTS,^22^ a picture is emerging in which even low‐density or recently cleared malaria infections are a significant contributor to invasive NTS. Finally, evidence that carriage of sickle cell trait (that protects from malarial anemia) reduces the risk of contracting invasive NTS^23^ and that intensive efforts in the last 15 years to reduce the prevalence and incidence of malaria across Africa have been accompanied by marked falls in the incidence of invasive bacteremia, and especially invasive NTS^24^, ^25^ serves to reinforce the clinical observations linking these two diseases and suggests a related underlying pathophysiology.

## 2 INTESTINAL AND INVASIVE NTS INFECTIONS

**Figure 1:**
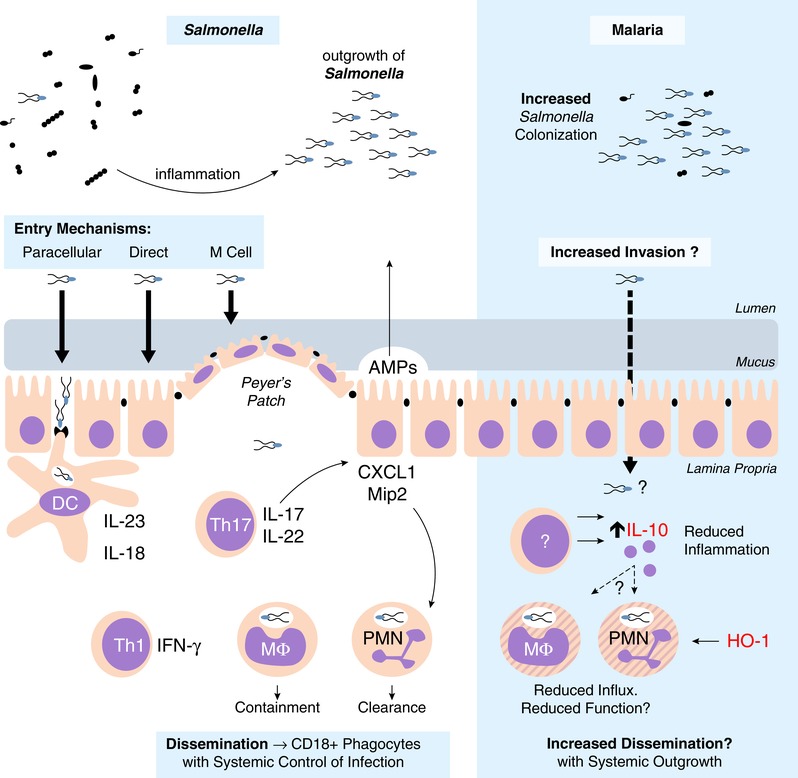
**NTS intestinal immune response**. NTS is a fecal‐oral pathogen, which thrives in the inflamed intestine. Tissue invasion in the distal intestine can be via direct invasion, uptake by M cells, or through paracellular spaces. Uptake by dendritic cells can initiate inflammation through the IL‐23/IL‐17 axis. Th17 cells promote neutrophil influx via the induction of neutrophil chemokines. NTS is able to persist within the Salmonella‐containing vacuole of macrophages, whereas neutrophils are efficient at NTS clearance. Systemic dissemination to draining lymph nodes is through CD18^+^ phagocytes. During experimental malaria, NTS colonization resistance is lowered, although it is unclear if there is an increase in tissue invasion. Regardless, inflammation (with reduced PMN influx) is reduced due to increased IL‐10 concentrations. However, the role of IL‐10, and potentially HO‐1, on intestinal neutrophil function and role for increased systemic dissemination are unclear

*Salmonella* can infect a broad host range (e.g., pigs, cattle, chickens, and humans) causing varying levels of damage, from enteric fever to severe gastroenteritis to asymptomatic carriage, depending on the particular serovar, typically defined by expression of LPS, flagellar, and capsular Vi antigens.^26^ With over 2500 known serovars,^27^ sterile immunity through natural infection or vaccination remains elusive.^28^, ^29^ The human‐restricted typhoidal serovars (*Salmonella* typhi and *Salmonella* paratyphi) are associated with systemic infection and carriage in the gallbladder^30^ but, intriguingly, these are not the serovars that are associated with malaria infections. Rather, malaria is associated with invasive disease caused by nontyphoidal serovars that can infect a broad range of different host species and are normally restricted to the intestine.^17^ Invasion of NTS through the intestinal mucosa can occur via their uptake by dendritic cells extruding dendrites between enterocytes into the intestinal lumen (paracellular uptake), via direct invasion of enterocytes or by passage through M cells of the Peyers Patches^31^ (Fig. 1), and this is dependent on a degree of inflammation.^32^, ^33^ Bacterial invasion triggers the IL‐23/IL‐18 inflammatory axis leading to T cell activation and production of inflammatory cytokines (including IFN‐γ, IL‐17, and IL‐22) and chemokines (CXCL1 and Mip2),^34^, ^35^, ^36^ eventually resulting in edema and infiltration of monocytes and neutrophils into the lamina propria,^37^ which are hallmarks of NTS pathology.

Although phagocytes, such as neutrophils and macrophages, are efficient in their uptake of NTS, the bacteria can disable the antibacterial machinery of macrophages to create *Salmonella* containing vacuoles (SCVs) within which they can persist and replicate.^38^ Proteins encoded within *Salmonella* pathogenicity island‐2 block lysosomal fusion allowing evasion of ROS‐mediated killing.^39^ Ultimately, clearance of bacteria from phagocytes is mediated by IFN‐γ, which induces breakdown of the SCV,^40^ releasing bacteria into the cytosol. Bacterial products now present in the cytosol can induce pyroptosis, a form of cell death involving both canonical and noncanonical inflammasome signaling with caspase‐1 and NLRP3 or caspase‐11 (in mice) and caspase‐4 and caspase‐5 (in humans), which lead to activation of IL‐1 and IL‐18.^41^, ^42^, ^43^ Bacteria released from disintegrating macrophages are cleared by neutrophils, a process that has been termed “phagocyte roulette.”^44^

Neutrophils, also called polymorphonuclear cells (PMNs), are terminally differentiated leukocytes with distinctive lobulated nuclei and contain antimicrobial cytoplasmic granules. PMNs are the most abundant white blood cell, with 1 × 10^11^ new cells emerging from the bone marrow daily. They are typically thought to have a very short lifespan in blood (∼7–24 hours^45^), although infection may delay apoptosis and increase lifespan.^46^, ^47^, ^48^ During infection and inflammation, PMNs are quickly mobilized to sites of injury. In the blood vessel, activated PMNs adhere to endothelium, extravasate and migrate along chemokine gradients to infectious foci.

**Figure 2:**
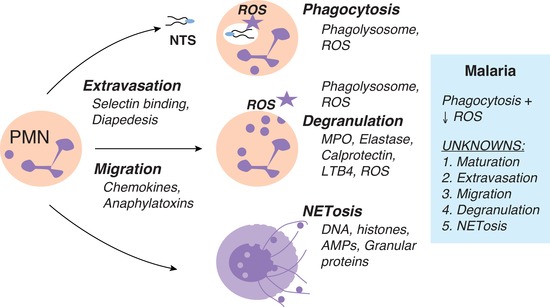
**Neutrophil function**. PMN extravasation is initiated by inflammatory mediators, which stimulate the upregulation of adhesion molecules, such as selectins, on endothelial cells allowing tethering and rolling of PMN. Chemokine gradients cause PMNs to “crawl” toward the site of infection until an endothelial junction is reached where diapedesis can occur. At the site of infection, neutrophils have numerous functions including phagocytosis, degranulation, and NETosis. Phagocytosis involves the ingestion of a pathogen into a phagolysosome containing granule proteins and ROS, to disable and digest internalized pathogens. Degranulation can also occur extracellularly where granular proteins, such as MPO and elastase, and ROS can be released from PMNs. A novel function of PMNs is NETosis, which is the release of NETs. These decondensed chromatin fibers decorated with histones and granular proteins trap and kill pathogens to prevent further dissemination. It is known that during malaria infection, neutrophils maintain the ability to phagocytose NTS; however, PMNs display impaired ROS production. Mechanisms still to be investigated include: maturation, extravasation, migration, degranulation, and NETosis

PMNs are professional phagocytes, which use receptor‐mediated phagocytosis to internalize pathogens and debris into phagolysosomes.^49^ Intracytoplasmic granules containing cathepsins, elastases, and myeloperoxidases fuse with the phagolysosome to digest internalized pathogens, in a process known as degranulation; leakage of granules or their contents into the extracellular milieu can be a significant cause of tissue damage during infection.^49^, ^50^ Release of reactive oxygen species (ROS), produced via an NADPH oxidase‐dependent process, into the phagolysosome is an additional, very important, bactericidal mechanism. PMNs can also kill extracellular pathogens by degranulation, secretion of ROS, or the release of neutrophil extracellular traps (NETs). NETs consist of externalized decondensed chromatin decorated with granular proteins and histones to prevent the dissemination of pathogens.^51^, ^52^ Serine proteases and histones provide antimicrobial activity against trapped pathogens. NETs also permit subsequent phagocytosis by proximate phagocytes (Fig. 2).

Although macrophages can be permissive to NTS growth, inflammatory monocytes and neutrophils are efficient in killing *Salmonella* through oxidative stress.^53^ In the intestine, both mucosa‐associated and luminal neutrophils engulf *Salmonella*.^54^ However, infiltrating PMNs also promote intestinal inflammation,^55^ thereby increasing the risk of bacterial invasion, and produce ROS, which can transform carbon sources such as thiosulfate in the intestinal lumen into tetrathionate and the microbial fermentation product 1,2‐propanediol, allowing NTS to outgrow the competing microbiota.^32^, ^33^ PMNs also release calprotectin into the intestinal milieu where it sequesters zinc, further restricting the growth of the intestinal microbiota.^56^ Therefore, although PMNs can limit bacterial growth and prevent overwhelming infection, NTS can exploit neutrophil‐mediated inflammation in the intestine to ensure its survival and eventual transmission.^53^, ^57^

## 3 PATHOPHYSIOLOGY OF NTS–MALARIA COINFECTIONS

Clinically, NTS infections in patients in Africa are not associated with overt diarrheal disease^58^ suggesting that underlying coinfection may ameliorate the intestinal inflammation typically associated with NTS. Evidence from coinfection models supports this idea (Table 1): intestinal inflammation is markedly reduced in both mice and macaques coinfected with malaria and NTS compared to animals infected with NTS alone and this is associated with reduced neutrophil influx and lower levels of IFN‐γ and IL‐17^59^ (Fig. 1). In mice, this reduction in intestinal inflammation is mediated by IL‐10,^59^ a potent anti‐inflammatory cytokine, which is essential for minimizing tissue damage during malaria infections (discussed in detail below). Although reducing intestinal inflammation might be expected to reduce the likelihood of invasion of NTS into the lamina propria and subsequent systemic dissemination, malaria infection has other, less beneficial consequences for the gut. In humans, acute malaria infection, in the apparent absence of gastrointestinal pathogens, is commonly associated with mild to moderate diarrhea, perhaps indicative of dysbiosis and or increased intestinal permeability.^60^, ^61^ In mice, malaria infection causes a dysbiosis, changing the composition of the microbiota and providing a foothold for colonization of the intestinal epithelium by NTS and *E. coli*.^62^ Increased colonization, taken together with increased gut permeability and reduced availability of neutrophils to control the bacterial infection, may explain why, 48 h after challenge with NTS, bacterial loads in the draining mesenteric lymph nodes are 100‐fold higher in malaria‐coinfected mice than in mice without malaria.^59^

**Table 1:** Animal models of malaria and salmonella coinfection

Reference	Animal (strain)	*Plasmodium spp*.	NTS strain (serovar)	Route of NTS challenge	NTS challenge after *Plasmodium*	Endpoint	NTS‐related outcome
Roux et al.^77^	Mus (CBA/J)	*P. yoelii nigeriensis*	IR715 (ATCC 14028)	Intragastric	Day 0	Day 5	Increased CFU in Spleen, liver, and Peyer's patch.
Cunnington et al.^63^	Mus (C57BL/6)	*P. yoelii* 17XNL	12023‐GFP	Intraperitoneal	Day 15	18 h	Increased CFU in blood, spleen, and liver.
Chau et al.^124^	Mus (CBA/J)	*P. yoelii* 17XNL	IR715 (ATCC 14028)	Intragastric	Day 10	Day 14	l‐Arginine & l‐Citrulline supplementation during P.y. reduces NTS burden in mesLN
Lokken et al.^64^	Mus (CBA/J)	*P. yoelii nigeriensis*	IR715 (ATCC 14028)	Intragastric	Day 10	Day 12, 14	Increased CFU in liver (day 12/14), blood (day 14)
	Mus (CBA/J)			Intraperitoneal	Day 10	Day 12, 13	Increased CFU in liver (day 12/13), blood (day 13)
	Mus (C57BL/6J)			Intragastric	Day 10	Day 12	No increase in CFU, reduced liver PMN chemokines
	Mus (C57BL/6J)			Intraperitoneal	Day 10	Day 12	No increase in CFU
	Mus (C57BL/6J il10^–/–^)			Intragastric	Day 10	Day 12	Reduces CFU in single & co‐infected, restored liver PMN chemokines
	Mus (C57BL/6J il10Rflx:LysMcre)			Intraperitoneal	Day 10	Day 12	IL‐10R^–/–^ myeloid cells; loss in increased liver, blood CFU
	Mus (C57BL/6J il10flx:LysMcre)			Intraperitoneal	Day 10	Day 12	IL‐10^–/–^ myeloid cells; loss in increased blood CFU
Mooney et al.^59^	*Macaca mulatta*	*P. fragile*	IR715 (ATCC 14028)	Ligated ilieal loops	Day 14, 15	8 h	Reduced intestinal inflammation to NTS
	Mus (CBA/J)	*P. yoelii nigeriensis*		Intragastric	Day 10	Day 12	Reduced intestinal inflammation to NTS, increased mesLN CFU
	Mus (C57BL/6J)				Day 10	Day 12	Reduced intestinal inflammation & PMN influx
	Mus (C57BL/6J il10^–/–^)				Day 10	Day 12	Restored intestinal inflammation & PMN influx in IL‐10^–/–^
Mooney et al.^125^	Mus (C57BL/6)	*P. yoelii* 17XNL	BRD509	Intravenous	Day 14, 28	Day 17, 31	Increased CFU in liver
	Mus (C57BL/6J‐Slc11a1^+/+^)			Intravenous	Day 14	Day 17	No increase in CFU
	Mus (CBA/J)			Intragastric	Day 14	Day 18	No increase in CFU
Mooney et al.^62^	Mus (C57BL/6J)	*P. yoelii nigeriensis*	IR715 (ATCC 14028)	Intragastric	Day 10	Day 11	Increased NTS colonization in feces
	Mus (C57BL/6J) ‐ Germ Free				Day 10	Day 11	Increased NTS colonization in feces with fecal donation from P. yoelii‐infected mice into germ‐free mice
Lokken et al.^79^	Mus (CBA/J)	*P. yoelii nigeriensis*	IR715 (ATCC 14028)	Intragastric	Day 10	Day 12, 14	Increased CFU in liver (day 14 only)
	Mus (C57BL/6J il10^–/–^)			Intraperitoneal	Day 10	Day 12	Reduced CFU in liver in IL‐10^–/–^

Whether increased NTS colonization or lymph node dissemination directly increases the risk of systemic spread is not yet clear but major defects in control of systemic NTS are evident in malaria coinfection: bacterial loads in blood, liver, spleen, and bone marrow after intraperitoneal injection of *Salmonella* Typhimurium (bypassing any intestinal contribution) were 1000–10,000‐fold higher in *Plasmodium yoelii‐*infected mice than in malaria‐uninfected mice.^63^, ^64^

## 4 HEMOLYSIS, HEME OXYGENASE‐1, AND NEUTROPHIL FUNCTION DURING MALARIA–NTS COINFECTION

**Figure 3:**
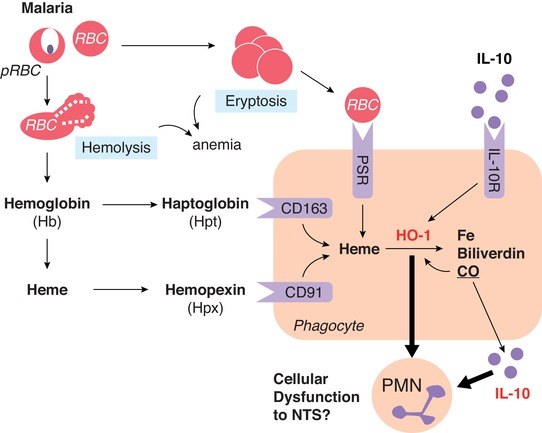
**Anemia, HO‐1, and IL‐10**. During malaria infection, anemia can occur through direct lysis of parasitized RBCs (hemolysis) or through eryptosis of uninfected RBCs—which are taken up by phagocytes through the phosphatidylserine receptor (PSR). Hemolysis results in release of hemoglobin (Hb), which can be further degraded to free heme. Serum scavenger proteins haptoglobin (Hpt) and hemopexin (Hpx) bind Hb and heme before uptake by phagocytes through CD163 and CD91, respectively. Intracellular heme is then degraded to iron, biliverdin, and carbon monoxide via the enzyme HO‐2 or the inducible isoform, HO‐1. HO‐1 can also be induced by IL‐10 receptor signaling. The impact of IL‐10 and HO‐1 during malarial anemia and hemolysis on phagocyte function remains poorly defined

Anemia is a heterogeneous condition defined as a reduction in circulating hemoglobin, the iron containing metalloprotein abundant in RBC, which ferry oxygen through the blood. One important cause of anemia is direct lysis of RBC (hemolysis) due to autoimmune conditions or infection. Some pathogens secrete hemolysins (e.g., *Staphylococcus spp*., *Streptococcus spp*., and *Clostridium spp*.) while other pathogens replicate inside, and ultimately lyse, RBC (e.g., *Babesia spp*., and *Bartonella spp*., and *Plasmodium spp*.). In malaria infection, direct destruction of RBC by parasitization is compounded by eryptosis of large numbers of uninfected RBC by processes that are still incompletely understood;^65^ however, complement‐mediated opsonization of bystander RBC has been noted.^66^, ^67^ Regardless of the mechanism, hemolysis results in the release of hemoglobin, or its breakdown product heme, into the plasma (Fig. 3). Free heme is prooxidant and highly cytotoxic, contributing to endothelial injury and hypertension.^68^, ^69^ Extracellular hemoglobin is scavenged by haptoglobin, which then binds to CD163 on the surface of macrophages and is internalized and degraded. Similarly, free heme is sequestered by hemopexin and internalized by binding to CD91. Intracellular heme is then degraded into equimolar amounts of iron, carbon monoxide, and biliverdin through the action of heme oxygenase (HO). Under homeostatic conditions, this process is mediated by the constitutively expressed HO isoform, HO‐2. However, under conditions of hypoxia, oxidative stress, or infection (e.g., presence of LPS), the inducible isoform HO‐1 (encoded by heme oxygenase‐1 gene [*hmox1*]) is upregulated to prevent heme‐induced pathology and ensure efficient iron recycling.^70^, ^71^ Importantly, plasma heme and HO‐1 concentrations are raised during both acute^72^ and subclinical^73^ *P. falciparum* malaria infections in humans, and during acute *P. yoelii* infection in mice,^63^ competitive inhibition of HO‐1 enzyme activity restores the ability of *P. yoelii*‐infected mice to control systemic NTS infections.^63^ Moreover, *hmox1* induction increases NTS growth in murine macrophages,^74^ indicating that hemolysis (via heme and HO‐1) rather than malaria, per se, may be the true risk factor for invasive NTS disease in malaria patients. In support of this notion, individuals with sickle cell anemia, which gives rise to periodic hemolytic crises, are also highly susceptible to invasive NTS disease^75^, ^76^; similarly, induction of acute hemolysis by antibody‐mediated RBC lysis^77^ or phenylhydrazine treatment^63^ also renders mice highly susceptible to NTS.

The first indication that hemolysis has a negative impact on neutrophil function, and thus that neutrophil dysfunction might underlie increased risk of invasive bacterial disease in people with malaria or other hemolytic diseases, came from mouse coinfection studies where circulating neutrophils from malaria‐infected and phenylhydrazine‐treated mice were shown to efficiently phagocytose *S*. Typhimurium but were unable to kill; viable bacteria persisted and, indeed, replicated inside neutrophils, which were severely deficient in ROS production.^63^ Of note, in vitro, heme pretreatment reduced phagocytosis of *E. coli* by human and murine neutrophils^78^ and high circulating heme during *P. falciparum* infection can reduce in vitro phagocytosis of *Salmonella* by neutrophils.^72^ The observation that treatment with a synthetic heme polymer, hemin, induced similar neutrophil defects and that these defects can be reversed by competitive inhibition of HO‐1, then provided a link among malaria, hemolysis, HO‐1, and neutrophil dysfunction.^63^ ROS‐defective neutrophils have also been observed in children with acute malaria; importantly, these defects persist for up to 8 weeks after treatment,^72^ perhaps explaining why children with recent (past) malaria infection remain susceptible to invasive NTS.^21^

*Plasmodium* infection also reduces neutrophil mobilization into infected tissues including blood^63^ and, as discussed above, intestine^59^ and liver^64^; infiltration of inflammatory monocytes into the liver is also impaired.^79^ Although this may be due in part to the anti‐inflammatory effects of IL‐10 (as described below), there are also cell‐intrinsic effects within developing neutrophils. Neutrophil precursors in the bone marrow (i.e., granulocyte macrophage progenitor cells) of malaria‐infected mice express HO‐1 and have unusual surface phenotypes (F4/80 and Gr‐1 expression).^63^ It has been shown that HO‐1 reduces neutrophil influx into the inflamed lung^71^ suggesting a causal relationship between HO‐1 and reduced neutrophil migration but much more work is needed to fully characterize neutrophil maturation and function during malaria infection and to determine the extent to which the altered phenotype is mediated by the heme/HO‐1 pathway.

## 5 THE ROLE OF IL‐10 IN MALARIA NTS COINFECTION

IL‐10 is a potent anti‐inflammatory cytokine and an important regulator of inflammation‐induced pathology,^80^ it is therefore no surprise that systemic IL‐10 concentrations are elevated in highly inflammatory diseases such as sepsis^81^, ^82^ and malaria.^83^, ^84^ More surprisingly, however, circulating IL‐10 is also elevated during mild/uncomplicated^83^, ^85^ and asymptomatic/subclinical malaria infections^73^, ^86^; indeed these cases may represent successful balancing of inflammation‐mediated parasite control and effective regulation of inflammation by IL‐10. In malaria, IL‐10 can come from both innate and adaptive immune cells, including Th1‐derived regulatory T cells that coproduce IFN‐γ and IL‐10,^87^, ^88^, ^89^ and plays an essential role in both adaptive humoral immunity (promoting differentiation of T‐bet^+^ germinal center B cells)^90^, ^91^ and limiting tissue damage.^92^, ^93^, ^94^

The anti‐inflammatory properties of IL‐10 include rendering phagocytes refractory to activation and/or directing macrophage polarization to a regulatory “M2" phenotype.^95^, ^96^ The impact of IL‐10 on neutrophil function is well described, reducing recruitment and migration in response to anaphylatoxins and ultimately bacterial clearance.^97^, ^98^ Further, neutrophils themselves can be a source of IL‐10, induced by regulatory T cells.^99^ During malaria NTS coinfection in mice, LysM‐expressing cells are a significant source of IL‐10 and its ablation reduces NTS bacteremia.^64^ IL‐10 suppresses neutrophil function through the activation of STAT3 and suppressor of cytokine signaling 3,^100^, ^101^ leading to the down‐regulation of IFN regulatory factor and NF‐κB family transcription factor.^102^ In addition, HO‐1 can be directly induced by IL‐10^103^ (Fig. 3). One hypothesis, therefore, is that malaria‐induced neutrophil dysfunction results from hemolysis‐ and IL‐10‐driven HO‐1 induction. In support of this hypothesis, in a recent study of persistent malaria infections, we have found that heme drives inflammation, that parasite density and inflammation then drives IL‐10 production, and that heme and IL‐10 both then induce HO‐1.^73^

## 6 COMPLEMENT DEPLETION DURING MALARIA

Complement proteins play an essential role in orchestrating inflammation and pathogen clearance,^104^, ^105^ including during *Salmonella* infection.^106^, ^107^ In brief, the classical pathway is activated by antibody (IgM and IgG) and the alternative pathway is activated by spontaneous hydrolysis of C3.^108^ C3 becomes membrane bound and cleaves C5, creating soluble C5a and membrane‐bound C5b.^109^ The complement anaphylatoxin C5a is a potent chemoattractant for neutrophils while also inducing expression of cell adhesion molecules on endothelial cells (such as P‐selectin) that permit neutrophil translocation into tissue.^110^ C5a also enhances neutrophil resistance to apoptosis,^111^ allowing the cells more time to perform effector functions. After bacterial uptake, maturation of the neutrophil phagosome requires its fusion with antimicrobial granules and the production of reactive superoxide anions (O_2_^–^) following G oxidase assembly on the phagolysosomal membrane.^112^ Assembly of NADPH oxidase is primed through the interaction of C5a and its receptor (C5aR and CD88).^113^, ^114^ Indeed, the *E. coli*‐induced oxidative burst in phagocytes is almost entirely dependent on CD88 signaling.^115^ Opsonization and killing of invasive NTS strains by neutrophils isolated from Malawian children require both antibodies and complement.^107^

**Figure 4:**
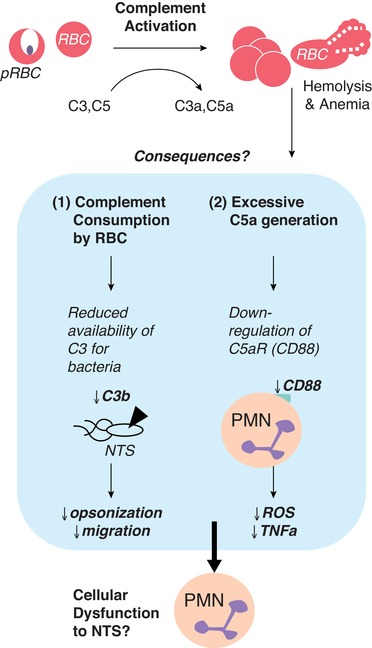
**Complement depletion**. During malaria infection, systemic complement activation occurs due to deposition on both infected and uninfected RBCs. This results in reduced concentrations of circulating C3 and C5 and generation of anaphylatoxins (C3a and C5a). The consequences that may impact PMN function are 2‐fold; (1) a reduction in C3 available for deposition on extracellular NTS leading to opsonization and migration and/or (2) excessive C5a reducing CD88 (C5aR) on neutrophils leading to reduced ROS and cytokine production. Additional work is needed to clarify the impact of complement activation and subsequent depletion during malaria on risk to NTS bacteremia

Complement activation on the surface of uninfected RBCs presumably increases the rate of turnover of RBCs during malaria infection,^116^ possibly due to reduced availability of complement regulatory proteins such as CD55.^117^, ^118^, ^119^ Complement‐mediated red cell lysis not only exacerbates the anemia associated with malaria, but will also increase circulating heme and therefore HO‐1 concentrations and reduce the availability of complement components,^120^ which may impair ability to control invasive bacterial infection (Fig. 4). Hypothetically, excess C5a in plasma may reduce CD88 on PMNs, and thus reduce the oxidative burst. Further, depletion of complement proteins (with concomitant reduction in expression of endothelial selectin) and reduced generation of anaphylatoxins could reduce neutrophil migration into infected tissues. Further studies are required to determine the significance of complement activation and complement consumption during malaria and risk of invasive NTS.

## 7 CONCLUDING REMARKS

We have provided a rational for considering that hemolysis and inflammation, leading to induction of HO‐1 and IL‐10 and activation of complement, during malaria infections might all contribute to the increased susceptibility to bacterial coinfection. It is likely that these pathways synergize to increase risk of invasive NTS: in malaria‐infected mice, both exogenous IL‐10 and anemia were required for increased bacteremia^64^; HO‐1 is induced both by heme and by IL‐10; and the carbon monoxide generated from heme catabolism can induce both HO‐1 and IL‐10.^121^, ^122^ Ultimately, it is important to define how these factors may converge to alter neutrophil biology. Through this, we may begin to understand if targeted treatment, or even prophylactic antimalarial treatment, can improve neutrophil function and so reduce the burden of invasive bacterial disease in malaria endemic populations. Given the importance of neutrophils in clearance of NTS, work is now needed to better describe the impact of malaria on this innate immune cell.

The risk of invasive NTS during malaria in sub‐Saharan Africa is well defined for acute malaria infection. However, the majority of malaria infections in the world are asymptomatic, with chronic, low‐density infections.^123^ As recent and low‐density malaria infections are a risk factor for NTS bacteremia,^21^ it is also important to understand if hemolysis, and resulting induction of HO‐1 and IL‐10, seen during these “asymptomatic” infections^73^ reaches the threshold needed for neutrophil dysfunction. In other words, in addition to defining the pathways leading to neutrophil dysfunction, we also need to identify the point at which the balance tips from these being host protective to increasing the risk to invasive NTS. Importantly, these pathways may contribute to severe bacterial disease even in the absence of malaria infections: in sepsis patients, we have observed that raised concentrations of heme, HO‐1, and IL‐10 are positively correlated with disease severity and mortality.^82^ Also, while “invasive” NTS is seen in immunocompromised hosts (such as those with malaria infection), it remains unclear if this is due to increased intestinal invasion, increased dissemination from draining lymph nodes, failure to control systemic bacterial replication, or a combination of any of these. Moreover, the extent to which this is primarily a neutrophil defect requires further exploration.
